# Reference ranges for the polyethylene glycol (PEG) precipitation activity (%PPA) of eight routine enzyme activities

**DOI:** 10.1016/j.plabm.2022.e00304

**Published:** 2022-12-17

**Authors:** Carmen Bürki, Martin Volleberg, Linnea Blomgren, Sean Froese, Martin Hersberger

**Affiliations:** a Medica Medizinische Laboratorien Dr. F. Kaeppeli AG, Zurich, Switzerland; b Division of Clinical Chemistry and Biochemistry, Children's Research Center, University Children's Hospital Zurich, University of Zurich, Switzerland; c Division of Metabolism and Children's Research Center, University Children's Hospital Zurich, University of Zurich, Switzerland

## Abstract

Macroenzymes are high-molecular weight forms of enzymes whose presence in human sera can lead to non-pathological, elevated enzyme activities, resulting in further unnecessary clinical evaluation. Precipitation with polyethylene glycol (PEG) is an efficient method for removing macroforms from patient samples and can therefore be used for their identification. Cut-offs (99. Percentiles) for the PEG precipitation activity (%PPA) for eight routine enzyme activities were determined on Abbott's Alinity c, namely: AST (61%), ALT (70%), GGT (41%), LDH (45%), lipase (56%), ALP (17%), CK (36%) and PAMY (45%). Two macroforms (PAMY and CK) were then identified by gel filtration chromatography. We suggest that a %PPA above the enzyme-specific cut-off makes the presence of a macroform possible while a %PPA ≥80%, i.e. markedly above the cut-off, makes it very likely for all enzymes.

## 1 Introduction

Macroenzymes are high-molecular weight forms (macroforms) of enzymes that result from post-translational modifications, which are most often associations of the free enzyme with immunoglobulin, i.e. autoantibodies (type 1 macroenzymes). Enzymes that form complexes with other plasma components or by self-polymerization are called type 2 macroenzymes [1,2]. Due to their high molecular weight, these immune complexes can persist in human plasma, leading to accumulation and consequently to increased enzyme activities [3]. Type 1 macroenzymes can be associated with some diseases but are most likely not pathological and therefore have diagnostic but not clinical relevance [1,4]. Hence, they lead to further investigations and thus unnecessary costs [1,5].

Many different serum enzymes can form macroforms [4], with this project focusing on aspartate transaminase (AST), alanine transaminase (ALT), gamma-glutamyl transferase (GGT), lactate dehydrogenase (LDH), lipase, alkaline phosphatase (ALP), creatine kinase (CK) and pancreatic amylase (PAMY). Thereby, macro-amylase is the most common and macro-CK the second most common form [2].

There are several methods for detecting macroforms, the simplest being precipitation with polyethylene glycol (PEG) [1,5]. Another way to determine the presence of macroforms is to separate them from the free enzymes using size exclusion chromatography (SEC). Through SEC, the high-molecular weight macroforms separate from the low-molecular weight free enzymes via column exclusion, resulting in an earlier elution [6]. The apparent molecular weight of type 1 macroforms approximate that of an immunoglobulin, which is around 150 kDa, plus twice the size of the free enzyme (ratio 1:2) [1,7].

The aim of this project was to evaluate cut-off values for the PEG precipitation activity (%PPA) on Abbott's Alinity c for each of the plasma enzymes mentioned. This allows a quick assessment of the presence of a suspected macroform.

## 2 Materials and methods

### 2.1 Materials

For precipitation experiments, a PEG solution (250 g/L) was prepared by dissolving PEG 6000 in 0.9% saline solution. Anonymized plasma samples from the routine Clinical Chemistry laboratory of the University Children's Hospital in Zurich as well as serum samples from the private laboratory medica Medizinische Laboratorien Dr. F. Käppeli in Zurich were used. Samples were selected randomly from the pool of elevated enzyme concentrations. They were stored in the fridge or freezer and were less than one year old before analysis. Serum and plasma samples (n = 211) were prepared by thawing, vortexing and centrifuging (1′500 g, 10 min) to receive homogenous, particle-free samples.

### 2.2 PEG precipitation

Enzyme activities (AST, ALT, GGT, LDH, lipase, ALP, CK, PAMY) in the samples were measured as blank, undiluted version (*initial*) and as 1:1-dilution in PEG solution (250 g/L, *PEG*). The diluted samples were vortexed (1 min), centrifuged (9′600 g, 10 min) and the supernatant was analyzed using Abbott's Alinity c. The method was adapted from Davidson et al. [5].

PEG precipitation activity was calculated as follows [5]:

$$\%\;PPA=\frac{Enzyme\;Activity\;\left(Blank\right)-Enzym\;Activity\;\left(PEG\right)*2}{Enzyme\;Activity\;\left(Blank\right)}*100$$

## 3 Results

A total of 211 samples were analyzed to calculate the reference range for the %PPA of AST, ALT, GGT, LDH, lipase, ALP, CK and PAMY. For the analysis, only results that were at least three times higher than the lower limit of quantification for the individual enzyme were included. This resulted in 51–131 PEG precipitation activity determinations per enzyme (Table 1). With these we calculated the 99. percentiles (Table 1) after excluding outliers (Supplementary).

**Table 1:** Median and 99. Percentiles of the %PPA of the enzymes AST, ALT, GGT, LDH, lipase, ALP, CK and PAMY are shown. Furthermore, upper reference values, outlier's enzyme activity, their %PPA and FPLC test result for a macroform are listed.

		AST	ALT	GGT	LDH	Lipase	ALP	CK	PAMY
Sample Size	n	109	51	119	67	61	137	122	106
Median	% PPA	16.4	38.3	8.30	16.9	30.5	0	5.40	20.0
99. Percentile		60.7	69.6	40.8	44.6	56.0	16.6	36.4	44.5
Upper ref. value (f/m)	U/L	35/50	35/50	40/60	250	60	105/130	170/190	53
Outlier	U/L	–	–	1641	–	155	982	83	259
Outlier	% PPA	–	–	45.8	–	57.3	17.3	83.1	93.8
FPLC	Macroform	–	–	–	–	No	No	Yes	Yes

**Fig. 1:**
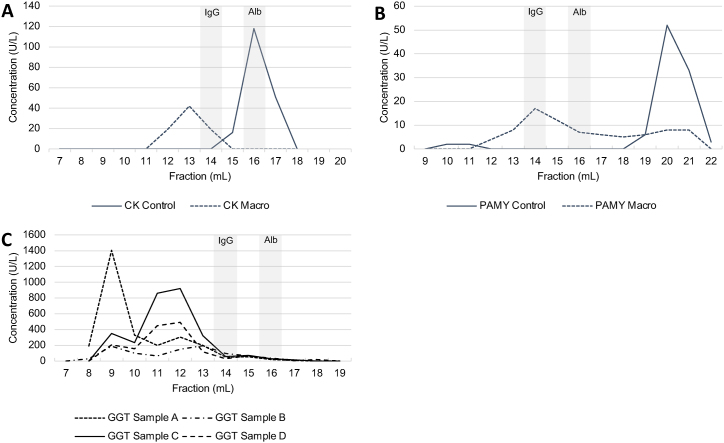
Gel filtration chromatograms of plasma samples analyzed for A) CK, B) PAMY, and C) GGT are shown.

All outlier samples (n = 4) were then tested for macroforms by separating the enzymes according to size using SEC. IgG and albumin eluted in fractions 14 and 16 mL, respectively, and served as size calibrators. This and four control samples with similar initial enzyme concentrations allowed us to identify one macroform for CK and one for PAMY (Fig. 1).

## 4 Discussion

The presence of macroenzymes in human sera can lead to elevated enzyme activities, which may result in further unnecessary and cost-intensive clinical evaluation. Methods for the detection of macroforms are well established, such as PEG precipitation and SEC [1,6]. In order to integrate a method for the identification of macroenzymes into routine analysis, reference values for the interpretation of PEG precipitation results on an individual analyzer should be available [5]. Here, we present the 99. percentiles for the %PPA of AST (61%), ALT (70%), GGT (41%), LDH (45%), lipase (56%), ALP (17%), CK (36%) and PAMY (45%) on Abbott's Alinity c. These results are comparable to those of Davison et al. [5], who found %PPA of 53%, 76%, 51%, 70%, 36%, 37% and 60% for AST, ALT, GGT, LDH, ALP, CK and PAMY, respectively. The differences in the reference ranges may be explained by the different clinical chemistry analyzers used (Abbott's Alinity c vs. Roche's Hitachi 717/Modular Multichannel Analyzers) and the number of samples analyzed (51–131 vs. approx. 40) [5,8]. Hence, when using analyzers other than Abbott's Alinity c, individual cut-off values should be evaluated.

To investigate whether the identified outliers of the %PPA were indeed all macroforms of the enzymes, we analyzed these samples by gel filtration chromatography. Of the four samples with presumed macroforms, we found one with a macro-CK and a macro-PAMY (Fig. 1A and B), while the other three samples did not have macroforms (data not shown). Interestingly, the originally measured CK activity (83 U/L) that showed the presence of the macroform was within the reference range, emphasizing that macroforms do not always lead to increased enzyme activity levels [9].

More challenging seems the definition of a macroform for GGT which normally exists in four different forms in human blood through its association with lipoproteins (type 2 macroforms), namely big-GGT (>2000 kDa), medium-GGT (940 kDa), small-GGT (140 kDa) and free GGT (70 kDa) [1,10]. Franzini et al.‘s findings and our analysis of four samples showed that most of the GGT is in the big to small-GGT fraction with almost no free GGT [10]. However, all four samples showed a different relative distribution of these large GGT forms hampering a clear identification of a specific macroform containing sample.

## 5 Conclusion

We defined the 99. percentile reference value for the %PPA for the enzymes AST, ALT, GGT, LDH, lipase, ALP, CK and PAMY and identified type 1 macroforms for PAMY and CK by SEC. We suggest, in accordance with a recent report [5], to consider that a %PPA above the 99. percentile for the specific enzyme makes the presence of a macroform possible while a %PPA ≥80%, i.e. markedly above the 99. percentiles, makes the presence of a macro-form very likely for all enzymes.

## Funding

This research did not receive any specific grant from funding agencies in the public, commercial, or not-for-profit sectors.

## CRediT author statement

**Carmen Bürki:** Conceptualization, Methodology, Investigation, Writing- Original draft preparation, Visualization. **Martin Volleberg:** Methodology, Investigation. **Linnea Blomgren:** Methodology, Investigation. **Sean Froese:** Methodology, Investigation, **Martin Hersberger:** Conceptualization, Methodology, Writing- Reviewing and Editing.

## Declaration of competing interest

The authors declare that they have no known competing financial interests or personal relationships that could have appeared to influence the work reported in this paper.

## Data Availability

Data will be made available on request.
