# Supplementary material for: Reference ranges for the polyethylene glycol (PEG) precipitation activity (%PPA) of eight routine enzyme activities
Source: Pract Lab Med. 2022 Dec 17;33:e00304. doi: 10.1016/j.plabm.2022.e00304 (PMC9792387; doi:10.1016/j.plabm.2022.e00304)
Supplement: Multimedia component 1 [file mmc1.zip › PEG_ShortCommunication_Supplementary.DOCX]

Short Communication Supplementary

**Reference Ranges for the Polyethylene Glycol (PEG) Precipitation Activity (%PPA) of Eight Routine Enzyme Activities**

Carmen Bürki^1^, Martin Volleberg^2^, Linnea Blomgren^3^, Sean Froese^3^, Martin Hersberger^2^

^1^medica Medizinische Laboratorien Dr. F. Kaeppeli AG, Zurich, Switzerland

^2^Division of Clinical Chemistry and Biochemistry, Children’s Research Center, University Children’s Hospital Zurich, University of Zurich, Switzerland

^3^Division of Metabolism and Children’s Research Center, University Children’s Hospital Zurich, University of Zurich, Switzerland

Materials and Methods

The polyethylene glycol (PEG) solution (250 g/L) was prepared by dissolving PEG 6000 (Molecular Biology Grade) from Merck (Darmstadt, Germany) in 0.9% saline solution from B. Braun (Sempach, Switzerland) and stirring at room temperature for 15 minutes.

To investigate the potential macroforms, gel filtration chromatography was performed using the fast protein liquid chromatography (FPLC) analyzer from Äkta Explorer (Pharmacia, Uppsala, Sweden) and a Superdex^®^ 200 10/300 GL gel filtration column from Cytiva (MA, USA). Tris-buffered saline solution (TBS) was used as running buffer which contained crystalline Tris base (Trizma^®^, ≥ 99.9%, 50 mM) and sodium chloride (NaCl, 150 mM) from Sigma-Aldrich (Buchs, Switzerland) dissolved in ultrapure water from the in-house ELGA Purelab Ultra water purification system (Labtec Service AG, Wohlen, Switzerland). The pH was adjusted to 7.5 by adding concentrated hydrogen chloride (HCl, conc.). The buffer was filtered through membrane filters (0.2 μm) from Thermo Scientific (NY, USA) before use.

The FPLC experiment was performed at 4 °C with a flow rate of 0.3 mL/min, a fraction size of 1.0 mL and an elution volume of 30 mL. Depending on the individual recorded chromatograms (UV, 280 nm) and the expected sizes of the enzymes, fractions between 7 and 22 mL were processed by concentration to 200 μL using Amicon® Ultra Centrifugal Filters (0.5 mL, 10 K) from Merck (Darmstadt, Germany) at 14’000 g for 5 min. Immunoglobulin G (IgG) and albumin (Alb) were used as calibrators (150 and 67 kDa, respectively).^7^ The method was adapted from Collins et al.**^6^**

Data Analysis

Data analysis was performed using Microsoft Excel for the evaluation of %PPA and MedCalc^®^ v14.8.1 (MedCalc Software Ltd, Ostend, Belgium) for the calculations of percentiles and determination of outliers (*Grubbs – right sided*).
